# Genome-Wide Identification of the TIFY Family in *Salvia miltiorrhiza* Reveals That SmJAZ3 Interacts With SmWD40-170, a Relevant Protein That Modulates Secondary Metabolism and Development

**DOI:** 10.3389/fpls.2021.630424

**Published:** 2021-02-18

**Authors:** Lin Li, Yuanchu Liu, Ying Huang, Bin Li, Wen Ma, Donghao Wang, Xiaoyan Cao, Zhezhi Wang

**Affiliations:** National Engineering Laboratory for Resource Development of Endangered Crude Drugs in Northwest of China, Key Laboratory of the Ministry of Education for Medicinal Resources and Natural Pharmaceutical Chemistry, College of Life Sciences, Shaanxi Normal University, Xi’an, China

**Keywords:** *Salvia miltiorrhiza*, jasmonate, TIFY proteins, SmJAZ3, SmWD40-170

## Abstract

*Salvia miltiorrhiza* Bunge (*S. miltiorrhiza*), a traditional Chinese medicinal herb, contains numerous bioactive components with broad range of pharmacological properties. By increasing the levels of endogenous jasmonate (JA) in plants or treating them with methyl jasmonate (MeJA), the level of tanshinones and salvianolic acids can be greatly enhanced. The jasmonate ZIM (JAZ) proteins belong to the TIFY family, and act as repressors, releasing targeted transcriptional factors in the JA signaling pathway. Herein, we identified and characterized 15 TIFY proteins present in *S. miltiorrhiza*. Quantitative reverse transcription PCR analysis indicated that the *JAZ* genes were all constitutively expressed in different tissues and were induced by MeJA treatments. SmJAZ3, which negatively regulates the tanshinones biosynthesis pathway in *S. miltiorrhiza* and the detailed molecular mechanism is poorly understood. SmJAZ3 acts as a bait protein to capture and identify a WD-repeat containing the protein SmWD40-170. Further molecular and genetic analysis revealed that SmWD40-170 is a positive regulator, promoting the accumulation of secondary metabolites in *S. miltiorrhiza*. Our study systematically analyzed the TIFY family and speculated a module of the JAZ-WD40 complex provides new insights into the mechanisms regulating the biosynthesis of secondary metabolites in *S. miltiorrhiza*.

## Introduction

The plant-specific TIFY family is characterized by a highly conserved motif (TIF[F/Y]XG) positioned within a TIFY domain of approximately 28-amino acids (aa) (Vanholme et al., 2007; Bai et al., 2011). According to phylogenetic and structural analyses, genes in that family can be assigned to four subgroups: TIFY, JAZ, PEAPOD (PPD), and ZIM-like (ZML). The TIFY subfamily proteins contain only a TIFY domain, whereas the ZML subfamily, including the ZIM and ZML proteins, contain a C2C2-GATA zinc-finger domain and a CCT domain (CONSTANS, CO-like, TOC1) (Staswick, 2008; Chung et al., 2009). In addition to the TIFY domain, the JAZ subfamily proteins are characterized by a conserved Jas motif of approximately 27 aa, near the C-terminal. These Jas sequences possess the characteristic SLX2FX2KRX2RX5PY motif and are similar to the N-terminal portion of the CCT domain (Staswick, 2008; Chung et al., 2009). In contrast, the PPD subfamily proteins possess a characteristic N-terminal PPD domain, and a modified Jas motif, that lacks the conserved PY (proline tyrosine) in the C-terminal region (Chung et al., 2009). Genes in the TIFY family have previously been systematically analyzed in several plant species, including 36 in *Brassica rapa*, 21 in *Brachypodium distachyon*, 34 in *Glycine soja*, 19 in grape (*Vitis vinifera*), 20 in rice (*Oryza sativa*), and 18 in *Arabidopsis* (Vanholme et al., 2007; Ye et al., 2009; Bai et al., 2011; Zhang et al., 2012, 2015; Zhu et al., 2013; Saha et al., 2016). The members of this family are involved in regulating diverse aspects of plant development, responses to abiotic stresses, and phytohormone treatments. For example, the *PvTIFY* gene plays a vital role in the adaptation of *Phaseolus vulgaris* to phosphorus (P) starvation by mediating JA signaling (Aparicio-Fabre et al., 2013). Certain *VvTIFY* genes in grapes can be induced by osmotic, low temperature, or drought, salinity conditions, as well as jasmonic acid (JA), or abscisic acid (ABA) treatments (Zhang et al., 2012). In rice, most *OsTIFY* genes are responsive to at least one type of abiotic stress, such as drought, salinity, or low temperatures (Ye et al., 2009). Perhaps the best-characterized members are the *JAZ* genes, which play a key role in the JA pathway (Chini et al., 2007; Thines et al., 2007; Yan et al., 2007; Garrido-Bigotes et al., 2019). Jasmonoyl-isoleucine (JA-Ile), the bioactive JA, is an important plant hormone that regulates various biological processes, including plant development, defense processes, and secondary metabolism (Creelman and Mullet, 1997; Liechti and Farmer, 2002; Farmer et al., 2003; De Geyter et al., 2012; Wasternack and Hause, 2013). Proteomic analysis of the JAZ interacting proteins under MeJA treatments in *Eleusine coracana*, illustrated that EcJAZ acts as a signaling hub for JA and other phytohormone signaling pathways (Sen et al., 2016). Moreover, JAZ expression, which regulates and fine-tunes the expression of downstream JA-responsive genes, is differential in various pathways and with certain stress responses (Demianski et al., 2011).

*Salvia miltiorrhiza* (*S. miltiorrhiza*), a model medicinal plant, is a well-known traditional Chinese herb (Ma et al., 2012; Xu et al., 2015). Its dried roots have been used to treat cardiovascular and cerebrovascular disorders, such as coronary heart disease, hyperlipidemia, and acute ischemic strokes (Su et al., 2015; Liu et al., 2016). *S. miltiorrhiza* has two primary active compounds namely water-soluble phenolic acids, including caffeic acid, rosmarinic acid, and salvianolic acid B (Liu et al., 2006), and lipid-soluble tanshinones containing dihydrotanshinone, cryptotanshinone, tanshinone I, and tanshinone IIA (Kai et al., 2011). These natural products can accumulate at low levels in plants over a long period (Ma et al., 2015). Both biotic and abiotic elicitors can induce the accumulation of secondary metabolites in *S. miltiorrhiza* (Zhao et al., 2010). Treatment with MeJA can lead to marked increase in tanshinones and salvianolic acids levels, as well as the expression of genes involved in their biosynthesis (Gu et al., 2012; Luo et al., 2015). However, it remains unclear how JA modulates the synthesis of these secondary metabolites in *S. miltiorrhiza*. The process of JA signal transduction occurs in three main stages: (1) The bioactive JA, JA-Ile is recognized by coronatine-insensitive 1 (COI1) protein forming Skp1/Cullin1/F-box protein COI1 (SCF^*COI*1^) complexes. (2) JAZ proteins are ubiquitinated by SCF^*COI*1^-type E3 ubiquitin ligase and degraded by the 26S proteasome. (3) MYC TFs are released thereby inducing the expression of downstream genes (Farmer, 2007). The JAs, SCF^*COI*1^ receptor complex, JAZ repressors, and TFs are all involved in JA signal transduction (Xie et al., 1998; Xu et al., 2002; Boter et al., 2004; Thines et al., 2007; Chini et al., 2010). JAZ is an important juncture that represses responses to JA by interacting with bHLH-TFs (MYC2, MYC3, MYC4, MYC5, GL3, EGL3, and TT8) and R2R3-MYB TFs (PAP, GL1, MYB21, and MYB24) (Fernández-Calvo et al., 2011; Song et al., 2011, 2013; Fonseca et al., 2014; Qi et al., 2014; Garrido-Bigotes et al., 2020). Certain JAZ proteins and their interaction partners have been identified in *S. miltiorrhiza*. SmMYC2a and SmMYC2b may interact with SmJAZ1 and SmJAZ2 to positively regulate tanshinones and salvianolic acid B production (Zhou et al., 2016; Yang et al., 2017). SmJAZ8 is a repressor involved in JA-induced biosynthesis of salvianolic acids and tanshinones via interactions with SmMYC2a (Ge et al., 2015; Pei et al., 2018). SmJAZ3 and SmJAZ9 negatively regulate tanshinones biosynthesis and JA signaling pathway in *S. miltiorrhiza* (Shi et al., 2016). SmJAZ9 can interact with AtMYC2, whereas SmJAZ3 cannot. In our study, we investigated the interaction partners of SmJAZ3 protein and molecular mechanism underlaying SmJAZ3 role in JA signaling pathway to regulate secondary metabolism.

WD40 repeat (WDR)-containing proteins feature a conserved sequence of approximately 40 amino acids, identified as the WD40 motif, which begins with glycine-histidine (Gly-His) and ends with tryptophan-aspartate (Trp-Asp) (Neer et al., 1994; Smith et al., 1999). Without a DNA-binding site, WD40 functions as a rigid scaffold for protein–protein and protein–DNA interactions, rather than directly regulating gene expression (Ramsay and Glover, 2005). Among the WD40 families, TTG1 has been the most studied regarding secondary metabolism. AtTTG1 and ortholog *TTG1* genes from *Zea mays*, *Medicago truncatula*, *Vitis vinifera*, and *Malus domestica* have been reported to be involved in the biosynthesis of anthocyanins or flavonoids (Walker et al., 1999; Carey et al., 2004; Pang et al., 2009; Matus et al., 2010; An et al., 2012). We have previously reported that SmTTG1 increased salvianolic acid B accumulations by forming the SmTTG1-SmMYB111-SmbHLH51 ternary transcription complex (Li et al., 2018). We also identified 225 *SmWD40* genes and analyzed the evolutionary relationship, gene structure, and conserved protein motif, which provided prelamination data for studying the function of SmWD40 in secondary metabolism (Liu et al., 2020).

In this study, we aimed to identify and characterize TIFY proteins present in *S. miltiorrhiza*. Phylogenetic trees, gene structures, and conserved motif analyses were conducted on the SmTIFY proteins. Further, a Y2H screening assay was used to elucidate the molecular mechanisms of SmJAZ3 in mediating JA signaling and secondary metabolism. The functions of its interaction partner, SmWD40-170, were then also identified and analyzed. We hypothesize that the molecular mechanism underlaying JAZ3-mediated regulation of secondary metabolism is via the JAZ-WD40 regulatory module in *S. miltiorrhiza*.

## Materials and Methods

### Plant Material

*Salvia miltiorrhiza* plants were acquired from Shangluo County, Shaanxi Province, China, and maintained in our laboratory at Shaanxi Normal University, Xi’an, China. Root, stem, leaf, and flower samples were collected from uniformly grown 2-year-old plants and immediately frozen in liquid nitrogen.

Tissue culture-derived plants of *S. miltiorrhiza* were used for the JA treatment experiments. After 30 days of culturing under normal laboratory conditions, the plants were assigned to two groups: (1) mock control, in which the leaves were sprayed with 10% ethanol; or (2) JA treatment, in which the leaves were sprayed with a solution of 100 μM MeJA plus 10% ethanol (Gu et al., 2012). Each experiment was performed three times (*n* = 3 per group) and whole plants were harvested from each group after 0.5 h, 1 h, 2 h, 4 h, 8 h, and 12 h. All samples were immediately frozen in liquid nitrogen and stored at −80°C until RNA extraction.

### Identification and Characterization of TIFY Family Genes and Phylogenetic Analyses

To identify all of the putative *SmTIFY* family members, we used the sequences for 18 *Arabidopsis TIFY* family genes, as well as 20 from rice, and 19 from grape, that were obtained from the TAIR databases^1^, TIGR databases^2^, and the Grape Genome Database^3^, respectively. Local BLASTs were conducted with the *S. miltiorrhiza* genome (Xu et al., 2016). The protein sequences database for *Arabidopsis*, rice, and grape TIFY proteins were used as queries for BioEdit. The SmTIFYs were determined by screening for the conserved TIFY domains using an NCBI conserved domain search^4^, Pfam^5^, HM-MER^6^, InterPro^7^, and SMART^8^ online tools. MEGA 6 software^9^ was used to investigate the phylogenetic relationships among TIFY proteins in the four species, based on the neighbor-joining algorithm and the bootstrap method (1000 replicates).

### Sequence Analysis of the SmTIFY Proteins

Relative molecular weights and isoelectric points (pI) of the TIFY family members were analyzed using ExPASy^10^. Subcellular localization of the proteins was determined according to WoLF PSORT^11^. The DNA and CDS sequences of the *SmTIFY* genes were submitted to the GSDS online tool^12^ to analyze their gene structures. The SmTIFY protein sequences were submitted to the MEME web server^13^ for analysis of the protein motifs. To explore potential *cis*-elements in the promoter sequences, 2000 bp of the SmTIFY genomic DNA upstream of the initiation codon (ATG) were downloaded, using the *S. miltiorrhiza* genome database. The promoter sequences were submitted to the Plant CARE database^14^ to predict the *cis*-acting elements.

### Expression Analysis of *SmTIFY* Genes in Different Tissues and Under MeJA Treatment

Total RNA was extracted from the *S*. *miltiorrhiza* root, stem, leaf, and flower samples. First-strand cDNA was reversed from the total mRNA, according to the instructions for the Prime Script^®^ RT Master Mix (Takara). RT-qPCR primer sequences were designed using Primer Premier 5.0, and a housekeeping gene (β*-Actin*) was used as an internal control (Supplementary Table 1). Several key genes encoding enzymes such as phenylalanine ammonialyase (*PAL*), cinnamate 4-hydroxylase (*C4H*), hydroxycinnamate-CoA ligase (*4CL*), tyrosine aminotransferase (*TAT*), hydroxyl phenylpyruvate reductase (*HPPR*), rosmarinic acid synthase (*RAS*), 1-deoxy-D-xylulose-5-phosphate synthase (*DXS*), 3-hydroxy-3-methylglutaryl-CoA reductase (*HMGR*), farnesyl diphosphate synthase (*FPPS*), geranylgeranyl diphosphate synthase (*GGPPS*), copalyl diphosphate synthase (*CPS*), and kaurene synthase-like synthase (*KSL*) were investigated using RT-qPCR (Supplementary Table 1). RT-qPCR analysis was conducted with SYBR Green (Takara Biotechnology) and a Roche LightCycler^®^ 96 real-time PCR machine. All experiments were performed with three biological replicates. Relative expression levels were calculated based on the 2^–△△*Ct*^ method (Vandesompele et al., 2002). Statistical significance was assessed using the Student’s *t*-test.

### Yeast Two-Hybrid (Y2H) Screening and Assay

The CDS and partial Jas domain of the SmJAZ3 were amplified with specific primers (Supplementary Table 2) and cloned into the pGBKT7 vector as bait, to search for interacting proteins. The autoactivation test of the SmJAZ3 in the yeast was conducted as previously described (Li et al., 2018). Based on the previously described protocols (Pan et al., 2017), the *S. miltiorrhiza* cDNA library expressed in the Y187 yeast cells was mated with the AH109 yeast cells expressing SmJAZ3, and then screened for the interacting partners of SmJAZ3.

The Y2H assay was conducted to confirm the interaction between SmJAZ3 and SmWD40-170. The CDS of SmWD40-170 was cloned into pGADT7 to fuse with the activation domain as the prey. According to the manufacturer’s protocol for the Matchmaker Gold Yeast Two-Hybrid System (Clontech), BD-SmJAZ3 and AD-SmWD40-170 fusion constructs were co-transformed into yeast strain AH109, using the lithium acetate method (Gietz and Schiestl, 2007), and yeast cells were grown on SD/-Leu/-Trp medium. Positive clones were then selected on SD/-Ade/-His/-Leu/-Trp medium with X-α-gal. The sequences for the primers used are listed in Supplementary Table 2.

### Bimolecular Fluorescence Complementation (BiFC) Assay

Based on the protocol from the Gateway technology manufacturer (Invitrogen, United States), the ORFs (without the termination codon) of *SmJAZ3* (*SmWD40-170*) were amplified from pMD19T-SmJAZ3 (pMD19T-SmWD40-170) with adaptor primers (Supplementary Table 2) and then cloned into the pDONR207 vector using a BP recombination reaction. For the BiFC assay, pDONR207-SmJAZ3 and pDONR207-WD40-170 were cloned into pEarleyGate202-YC and pEarleyGate201-YN, respectively, using the LR recombination reaction (Earley et al., 2010). Equal concentrations of the YN and YC recombinant plasmids were mixed before co-transformation. Co-transformed YC-SmJAZ3/YN and YN-SmWD40-170/YC served as negative controls. Then, the mixed plasmids were bombarded into onion epidermal cells, using particle bombardment with the Gene Gun PDS-1000, and then incubated at 28°C for 24 h. The fluorescence signals were detected using a Leica DM6000B microscope (Leica, Germany) at an excitation wavelength of 475 nm.

### Determination of Salvianolic Acids and Tanshinones Concentrations by Liquid Chromatography/Mass Spectrometry (LC/MS) Analysis

Transgenic lines were obtained by *Agrobacterium*-mediated transformation method (Yan and Wang, 2007). Three OE lines (OE-3, OE-7, and OE-8) and three RNAi lines (i-11, i-14, and i-15) were acquired from our laboratory and used for further studies (Liu et al., 2020). Two-month-old culture seedlings were first cultivated in hydroponic cultures for 7 days and then transplanted into the soil medium (perlite: vermiculite: grass ash = 1:1:3). After 2 months of cultivation, the roots were removed, washed, dried in an oven at 30°C, and then ground to a powder. The salvianolic acids and tanshinones compounds were extracted, as previously described (Wang et al., 2018).

Separation of the lipophilic tanshinone and hydrophilic salvianolic acid was performed using a conventional Welch Ultimate XB-C18 column with two ion monitoring modes (2.1 × 150 mm, 3 μm, Agilent Corporation, MA, United States) and the following conditions: mobile phase A, acetonitrile; mobile phase B, 0.1% formic acid; injection volume, 5 μL; flow rate, 0.4 mL/min; gradient elution conditions, 0 min (25% A) → 5 min (10% B); ion source, AJS (Agilent jet) and ESI (electrospray ionization); quantitative detection, multiple reaction monitoring (MRM) mode. The negative ion mode detection of salvianolic acid B and rosmarinic acid had the following properties: salvianolic acid B detection range (m/z), 717 519; rosmarinic acid detection range (m/z), 359 → 161; fragment voltage, 130 V; collision energy, 20 eV. The ion pattern test for the tanshinone IIA had the following properties: tanshinone IIA detection range (m/z), 295.1 → 277.1, fragment voltage of 140 V, and collision energy of 32 eV. The ion pattern test for cryptotanshinone had the following properties: cryptotanshinone detection range (m/z), 297.1 → 254.1, fragment voltage of 140 V, and collision energy of 26 eV. The standards and samples were tested according to the above conditions, and standard curves were constructed. The peak area measured with the standard solution was the ordinate, while the standard concentration was the abscissa. Regression equations and linear coefficients were then calculated.

## Results

### Identification and Characterization of the *TIFY* Family Genes in the *S. miltiorrhiza* Genome

**FIGURE 1:**
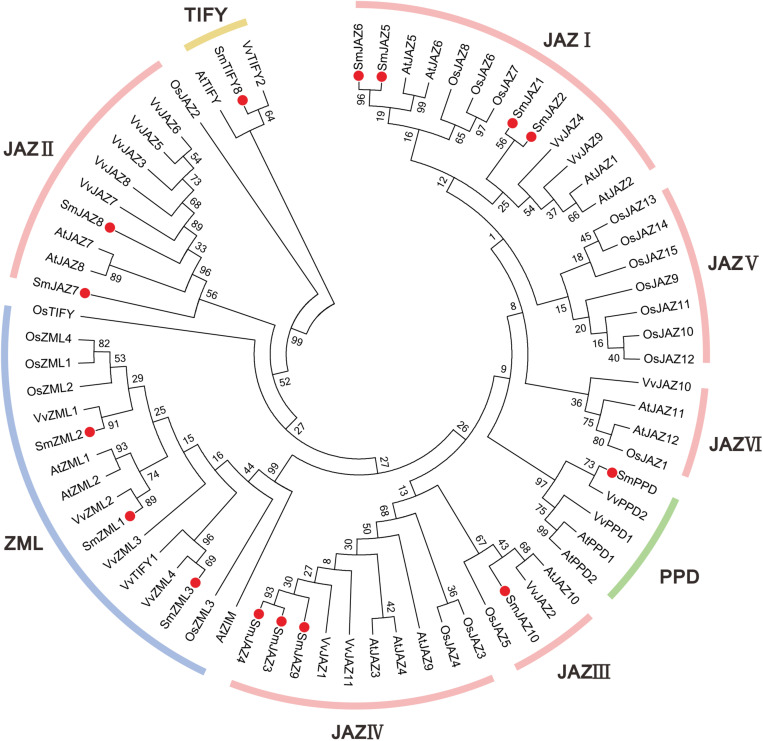
Neighbor-joining phylogenetic tree of TIFY proteins from *Salvia miltiorrhiza*, *Arabidopsis*, *Vitis vinifera*, and *Oryza sativa*. The tree was developed using MEGA 6 software and the bootstrap method (1000 replicates).

In additions to the four *SmJAZ* genes that we have previously reported, a total of 15 *SmTIFY* genes (1 *TIFY*, 1 *PPD*, 3 *ZML*s, and 10 *JAZ*s) were identified in the *S. miltiorrhiza* genome (Table 1) (Ge et al., 2015; Xu et al., 2016). These were named according to the existing numbering system used for the phylogenetic tree of *Arabidopsis* (Figure 1). The lengths of the SmTIFY amino acids ranged from 122 to 455, which is higher than that of other species. Subcellular localization analysis indicated that 10 of the SmTIFY proteins were nuclear, while SmJAZ7, SmJAZ8, and SmJAZ10 were located in the chloroplasts, and SmJAZ1 and SmJAZ6 in the cytoplasm and mitochondria, respectively.

**TABLE 1:** The basic information of *SmTIFY* family genes.

Gene ID	Accession no.	Genomic length (bp)	CDS length (bp)	Protein length (aa)	Molecular weight (Da)	pI	Sub-cellular localization
*SmJAZ1*	JQ936590	700	543	180	19815.67	9.14	Cyto
*SmJAZ2*	KX814385	855	642	213	22501.41	7.86	Nucl
*SmJAZ3*	KY225688	3367	1011	336	35515.09	9.16	Nucl
*SmJAZ4*	KY225684	2184	945	314	32463.59	9.38	Nucl
*SmJAZ5*	KY225689	4890	1368	455	49022.40	9.57	Nucl
*SmJAZ6*	KC864779	1968	705	234	25417.54	9.51	Mito
*SmJAZ7*	KX814384	962	372	123	14681.92	10.34	Chlo
*SmJAZ8*	JQ936591	557	369	122	13766.72	9.69	Chlo
*SmJAZ9*	KX814388	3129	921	306	32428.60	8.28	Nucl
*SmJAZ10*	KX814383	1902	540	179	19637.53	9.45	Chlo
*SmTIFY8*	KX814386	3427	1119	372	39330.10	8.82	Nucl
*SmPPD*	KX814387	4107	828	275	30085.00	5.79	Nucl
*SmZML1*	KY225685	2930	894	297	32564.05	6.06	Nucl
*SmZML2*	KY225686	2627	918	305	33295.74	5.54	Nucl
*SmZML3*	KY225687	4027	981	326	35776.06	5.07	Nucl

*Cyto, cytoplasmic; Nucl, nuclear; Mito, mitochondrial; Chlo, Chloroplast.*

### Phylogenetic Analysis of the TIFY Family Members

To investigate the evolutionary patterns and phylogenic relationships among the TIFYs in *S. miltiorrhiza* (15 proteins), grape (19 proteins), rice (20 proteins), and *Arabidopsis* (18 proteins), a neighbor-joining phylogenetic tree was constructed (Figure 1). All proteins fell into four major groups: TIFY, PPD, ZML, and JAZ. Among them, the ZML/ZIM proteins from all four species were clustered together. The TIFY proteins, which contain only a TIFY domain, were clustered into a single branch, except for OsTIFY. The PPD subfamily members comprised only AtPPD, VvPPD, and SmPPD. As expected, the JAZ subfamily members accounted for most proteins, including all the AtJAZ, VvJAZ, SmJAZ, and some OsTIFY (putative OsJAZ) proteins. SmJAZ proteins were clustered into four layers (JAZ I, JAZ II, JAZ III, and JAZ IV). SmJAZ1, SmJAZ2, SmJAZ5, and SmJAZ6 were assigned to JAZ I; SmJAZ7 and SmJAZ8, to JAZ II; and SmJAZ3, SmJAZ4, and SmJAZ9, to JAZ IV; JAZ III contained only one of these proteins, SmJAZ10.

### Sequence Analysis of the SmTIFY Family

**FIGURE 2:**
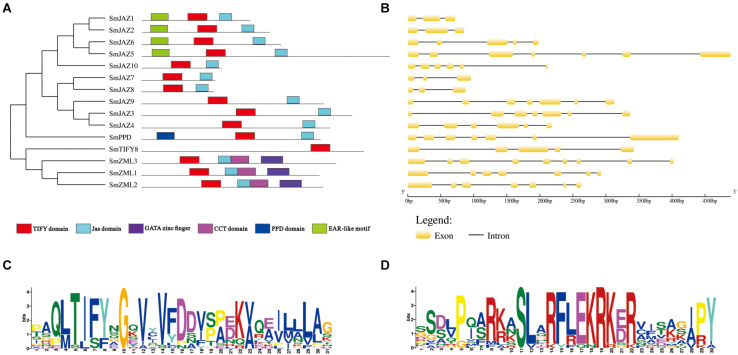
**(A)** Distribution of conserved domains within SmTIFY, SmJAZ, SmPPD, and SmZML proteins. Relative positions of domains within each protein are shown in different colors. **(B)** Gene structures of *SmTIFY* gene family. Exon, yellow-filled boxes; intron, black single lines. **(C)** Sequence logos of TIFY domains from SmTIFY proteins. **(D)** Sequence logos of Jas domains from SmTIFY proteins.

The full lengths of the CDSs with their corresponding genomic DNA sequences were compared to determine the number and positions of the exons and introns. Each *SmTIFY* gene has three to nine exons (Figure 2B). The structures in this family are diverse, especially within the SmJAZ subfamily. To identify the distribution of the conserved domains and the multiple sequence alignments among the SmTIFY proteins, we examined their sequences. The results from our MEME analysis showed that members of this family have six putative conserved domains, namely the TIFY domain, Jas domain, GATA zinc finger, CCT domain, PPD domain, and EAR-like motif (Figure 2A). In general, the TIFY domain contains 31 amino acids, with a highly conserved pattern of TIFYXG, T [L/I] SFXG, and SLSFQG (Figure 2C). All SmTIFY proteins include a TIFY domain, while all SmJAZ proteins have a Jas domain, with the conserved motif SLX2FX2KRX2RX5PY (Figure 2D). In addition, the N-terminals of SmJAZ1, SmJAZ2, SmJAZ5, and SmJAZ6 have EAR-like motifs. Three of the SmZML proteins (SmZML1, SmZML2, and SmZML3) contain a TIFY domain, Jas domain, CCT domain, and GATA zinc finger. The N-terminal of the SmPPD also has a PPD domain. In contrast to all other members, SmTIFY8 only has a TIFY domain.

### Analysis of the *Cis*-Elements in the SmTIFY Family

A search of the PlantCARE database showed the promoter sequences of the SmTIFYs. Among them, were a series of *cis*-elements that are involved in responses to light, biotic and abiotic stresses, phytohormones (ABA, MeJA, auxin, SA, gibberellin, and ethylene), circadian rhythms, and fungal elicitors (Supplementary Table 3). We also identified TF binding sites, such as an MBS and G-box. Almost all members, except *SmPPD* and *SmZML1*, contained TC-rich repeats that are involved in plant defenses and stress responsiveness. All these genes, except *SmJAZ1*, *SmTIFY8*, and *SmZML2*, also have TCA-elements that are responsive to SA. *SmJAZ1*, *SmJAZ2*, *SmJAZ5*, *SmJAZ6*, *SmJAZ7*, and *SmZML2* have *cis*-elements involved in responses to MeJA. Except for *SmJAZ1* and *SmZML2*, all genes contain a HSE and MBS in their promoter sequences, and, except for *SmJAZ3*, have a G-box, which is a possible MYC2-binding motif (Yu et al., 2016). However, only *SmJAZ10* has C-repeat/DRE and WUN-motif elements that are involved in responses to cold, dehydration, and wounding. MBSI, an MBS that helps regulate genes for flavonoid biosynthesis, was only found in the *SmPPD* promoter sequence. All these *cis*-elements have essential roles in modulating gene expression, by controlling promoter efficiency. Therefore, these results provide vital information for further research into the functions of SmTIFY genes.

### Expression Analysis of the *SmTIFY* Genes

**FIGURE 3:**
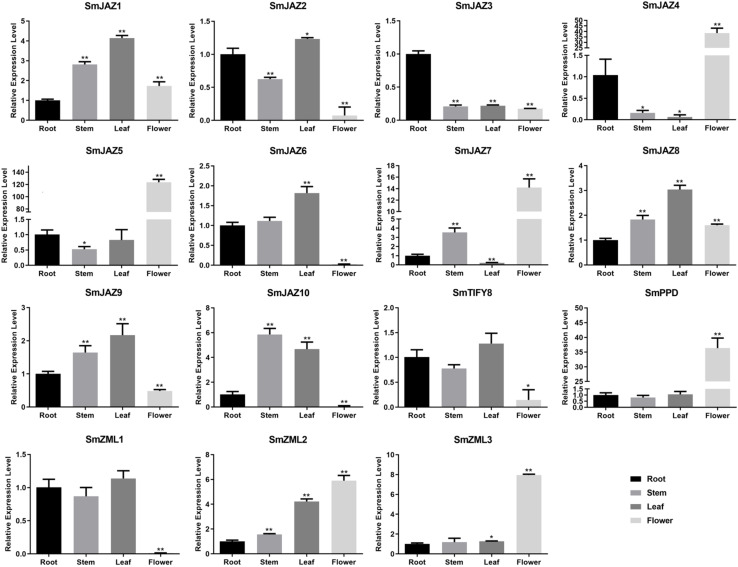
Relative expression of *SmTIFY* genes in root, stem, leave, and flower. All data represent averages of three biological replicates, error bars indicate SD. Statistical significance was determined using the Student’s *t*-test (*p < 0.05, **p < 0.01) between root, stem, leaf, and flower.

**FIGURE 4:**
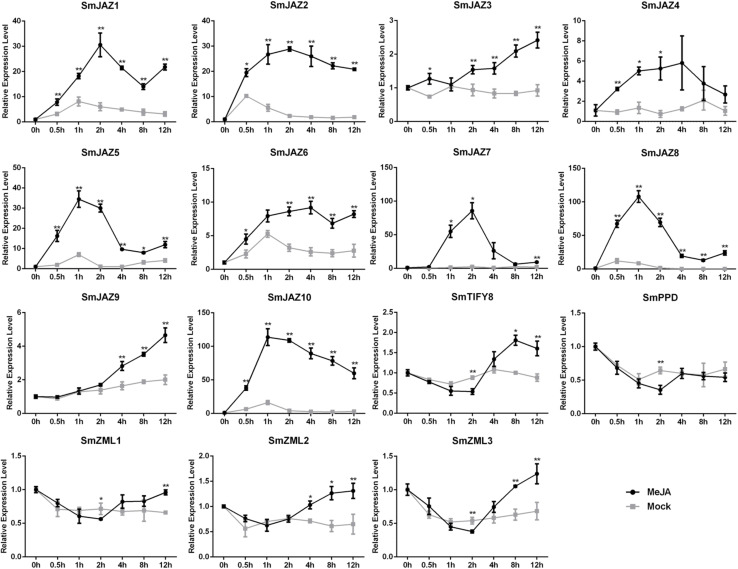
Relative expression level of *SmTIFY* genes in *S. miltiorrhiza* plants treated with mock control and 100 μM MeJA. All data represent averages of three biological replicates, error bars indicate SD. Statistical significance was determined by the Student’s *t*-test (*p < 0.05, **p < 0.01).

To determine the function of the *SmTIFY* genes, we monitored the expression of these genes in four tissue types sampled from *S. miltiorrhiza* (Figure 3). The most highly expressed genes were as follows: *SmJAZ1*, *SmJAZ6*, *SmJAZ8*, and *SmJAZ9* in the leaf; *SmJAZ4*, *SmJAZ5*, *SmJAZ7*, *SmPPD*, *SmZML2* and *SmZML3*, in the flower; and *SmJAZ3* was predominantly expressed in the root. Expressions of *SmJAZ2*, *SmJAZ6*, *SmJAZ10*, *SmTIFY8*, and *SmZML1* were lower in the flower tissues, while the expressions of *SmJAZ4* and *SmJAZ7* were lower in the leaf tissues. Interestingly, the expression patterns of *SmJAZ1* and *SmJAZ8* were similar in different tissues. However, none of the genes in the same subfamily showed similar expression patterns, which indicates that each member plays an irreplaceable role. To investigate the role of the *SmTIFY* family genes in the JA signaling pathway, we monitored their expression in response to exogenous MeJA (Figure 4). Most genes were induced within 2 h of treatment and their expression continued to increase over time. In particular, the peak in expression of *SmJAZ3*, *SmJAZ4*, and *SmJAZ9* was delayed compared with that of the other *SmJAZ* genes. In contrast, MeJA inhibited the expression of *SmTIFY8*, *SmPPD*, and *SmZML1* at 2 h, but delayed the response of *SmZML2* and *SmZML3*.

### Y2H Screening

Since the JA-responsive gene *SmJAZ3* negatively regulates tanshinones biosynthesis in *S. miltiorrhiza* (Shi et al., 2016), we selected *SmJAZ3* as a candidate gene, to explore the molecular mechanisms underlaying the regulation of JA-mediated secondary metabolism of *S. miltiorrhiza*. To determine whether SmJAZ3 affects potential target proteins or TFs, we screened for interacting proteins using a Y2H system. Initially, no autoactivation of the SmJAZ3 baits was detected (Supplementary Figure 1C). Since the results were not satisfactory when the full-length SmJAZ3 was used as the bait protein for the library screening, we used the Jas domain as the bait. The screening for the cDNA library in *S. miltiorrhiza*, resulted in 36 candidate proteins being identified, that interacted with SmJAZ3-Jas (Supplementary Figure 1F and Supplementary Table 4). Function annotation suggested that these candidate interaction proteins including JA signal member MYC2, as well as some enzymes are associated with biosynthesis process, metabolism process, and stress resistance (Supplementary Table 4). Interestingly, a WD40 protein, SmWD40-170 (Gene ID: ATA66299), was among them. The full-length CDS of the *SmWD40-170* was 972 bp, and it encoded a protein of 323 amino acids. It was located in the nucleus and the cytoplasm and is predicted to respond to MeJA-responsive elements (Liu et al., 2020). In addition, the SmWD40-170 responded to drought stress and regulated ABA- and H_2_O_2_-induced stomatal movements in the *S. miltiorrhiza* (Liu et al., 2020).

### Interactions Between the SmJAZ3 and SmWD40-170 Proteins

**FIGURE 5:**
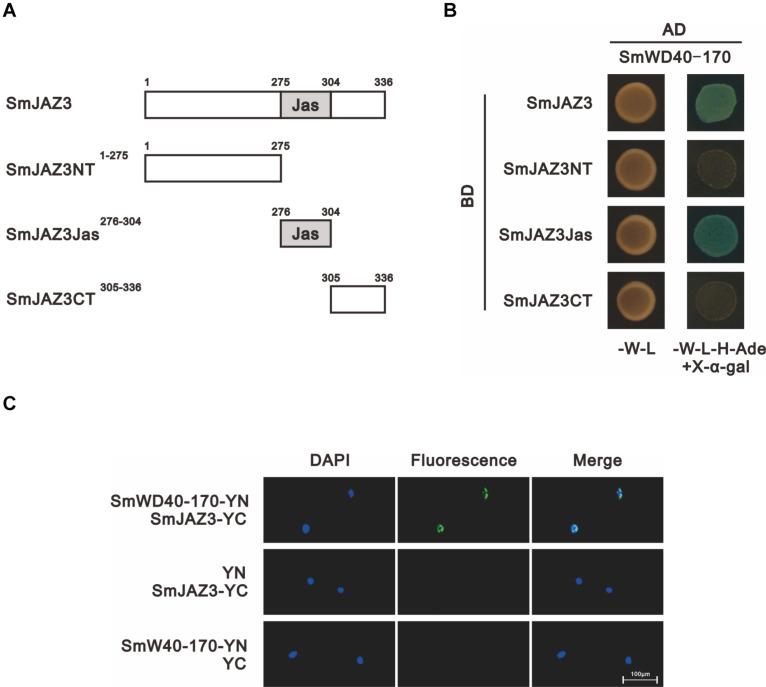
SmJAZ3 interacts with SmWD40-170. **(A)** Schematic diagrams show domain constructs of SmJAZ3. **(B)** Y2H assays was used to test the interactions of SmWD40-170 with different domains of SmJAZ3. **(C)** BiFC assays was used to detect the interaction between SmJAZ3 and SmWD40-170.

We analyzed the interactions between the SmJAZ3 and SmWD40-170 proteins. For the Y2H, except for the full-length sequence, the SmJAZ3 was divided into three parts: N-terminal fragment (amino acids 1 to 275), Jas motif fragment (amino acids 276 to 304), and C-terminal fragment (amino acids 305 to 336), to examine whether other domains of the SmJAZ3 were responsible for the interaction with the SmWD40-170 protein (Figure 5A). The results showed that the full-length and Jas motif of the SmJAZ3 interacted with the SmWD40-170 in yeast (Figure 5B). A BiFC assay was then used to examine the Y2H results. SmWD40-170 and SmJAZ3 were fused with the N-terminal and C-terminal, respectively, of the YFP. As expected, a strong fluorescent signal was detected in the nucleus when SmWD40-170 and SmJAZ3 were co-transformed into onion epidermal cells, whereas no fluorescent signal was observed in the control groups (Figure 5C). Taken together, our results suggest that SmJAZ3 could interact with SmWD40-170.

### SmWD40-170 Affects the Biosynthesis and Accumulation of Secondary Metabolites in *S. miltiorrhiza*

**FIGURE 6:**
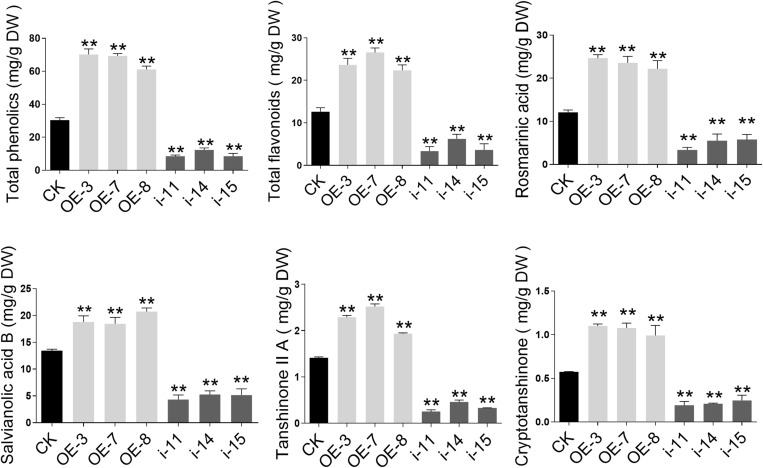
Secondary metabolites contents in control and SmWD40-170 transgenic roots of *S. miltiorrhiza*. Comparisons of total phenolic acid, total flavonoids, rosmarinic acid, salvianolic acid B, tanshinone IIA, and cryptotanshinone concentration among transgenic and control lines. All data represent averages of three biological replicates, error bars indicate SD. Statistical significance was determined using the Student’s *t*-test (**p < 0.01).

The levels of the total phenolic acids and total flavonoids in the OE-3, OE-7, and OE-8 lines, as well as in the i-11, i-14, and i-15 RNAi lines, were determined using the Folin–Ciocalteu method and the sodium nitrite-aluminum chloride colorimetric method (Dewanto et al., 2002; Chew et al., 2009) with 2-month-old roots of *S. miltiorrhiza*. Compared to the CK, the total phenolic acids and total flavonoids contents increased by more than 2 and 1.5 times, respectively, in the three OE lines. In the three RNAi lines, especially i-14, the contents of both total phenolic acids and total flavonoids were significantly decreased by 59.47% and 50.63%, respectively (Figure 6).

To further understand the effects of SmWD40-170 on the biosynthesis and accumulation of secondary metabolites in *S. miltiorrhiza*, the active components of salvianolic acids and tanshinones were more accurately determined and analyzed using LC/MS (Supplementary Figure 2). In comparison to CK, the concentrations of salvianolic acids and tanshinones were significantly increased in the OE-3, OE-7, and OE-8 lines (Figure 6). The rosmarinic acid content increased by 2.04 times, at most, and the salvianolic acid B content increased by approximately 1.54 times in the three OE lines, while the two tanshinones compounds increased by 1.37–1.78 times for tanshinone IIA and 1.72–1.91 times for cryptotanshinone, respectively. Compared to the CK, the levels of the three interference lines were the opposite, as was expected, and the contents of the four detected secondary metabolites were reduced by more than half. In particular, in the i-11 interference line, rosmarinic acid, salvianolic acid B, tanshinone II A, and cryptotanshinone were reduced by 71.93%, 67.95%, 82.23%, and 66.83%, respectively.

**FIGURE 7:**
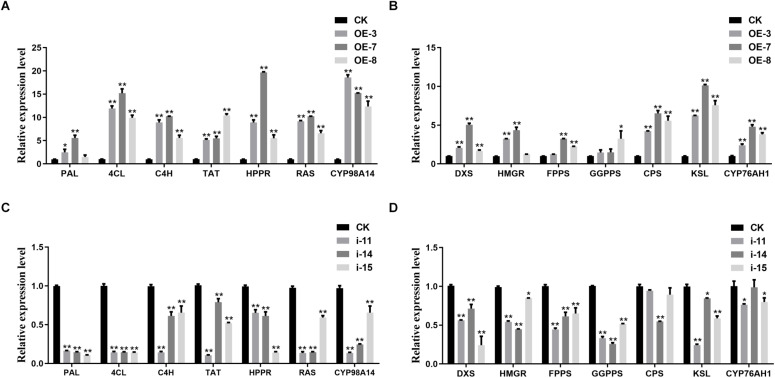
Expression analysis of salvianolic acids and tanshinones biosynthesis genes. RT-qPCR analyses of the key enzyme genes of salvianolic acids biosynthetic pathway in OE **(A)** and RNAi **(C)** lines. RT-qPCR analyses of the key enzyme genes of tanshinones biosynthetic pathway in OE **(B)** and RNAi **(D)** lines. All data represent averages of three biological replicates, error bars indicate SD. Statistical significance was determined using the Student’s *t*-test (*p < 0.05, **p < 0.01). *PAL*, phenylalanine ammonialyase; *C4H*, cinnamate 4-hydroxylase; *4CL*, hydroxycinnamate-CoA ligase; *TAT*, tyrosine aminotransferase; *HPPR*, hydroxyl phenylpyruvate reductase; *RAS*, rosmarinic acid synthase; *DXS*, 1-deoxy-D-xylulose-5-phosphate synthase; *HMGR*, 3-hydroxy-3-methylglutaryl-CoA reductase; *FPPS*, farnesyl diphosphate synthase; *GGPPS*, geranylgeranyl diphosphate synthase; *CPS*, copalyl diphosphate synthase; *KSL*, kaurene synthase-like synthase.

To determine whether metabolite accumulation was caused by changes in enzyme gene expression in the metabolic pathway, RT-qPCR was used to detect the expression of key enzyme genes involved in the salvianolic acids and tanshinones biosynthesis pathways in different lines. In the salvianolic acid biosynthesis pathway, the expression levels of *SmTAT*, *SmHPPR*, *SmPAL*, *SmC4H*, *Sm4CL*, *SmRAS*, and *SmCYP98A14* in the OE lines were significantly upregulated compared to the CK, but the activities of all the enzymes were repressed in the three interference lines (Figures 7A,C). In the tanshinones biosynthesis pathway, several enzyme genes, such as *SmDXS*, *SmHMGR*, *SmFPPS*, *SmGGPPS*, *SmCPS, SmKSL*, and *SmCYP76AH1* were markedly induced in the OE lines. In contrast, the expression levels of these genes were decreased in the interference lines compared to those in the CK (Figures 7B,D).

### Morphological Differences Between Transgenic Lines of SmWD40-170 in *S. miltiorrhiza*

**FIGURE 8:**
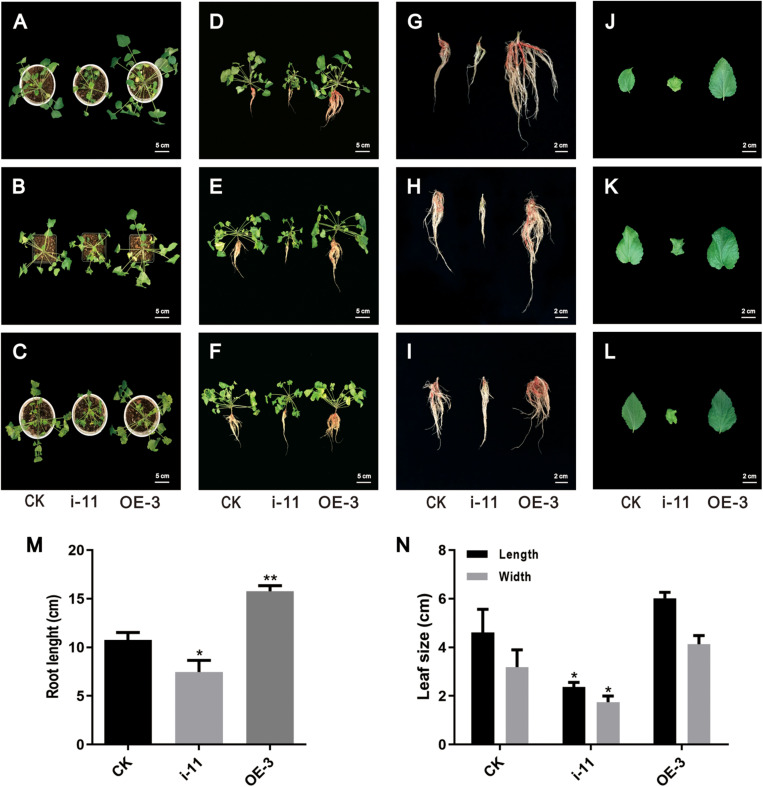
Morphological differences in the control and SmWD40-170 transgenic lines. **(A/B/C,D/E/F,G/H/I,J/K/L)** represent three independent repeats. **(A–F)** Control and transgenic seedlings cultured in soil for 2 months. **(G–I)** Control and transgenic roots. **(J–L)** Control and transgenic leaves. **(M)** Comparison of the root length in control and transgenic lines. **(N)** Comparison of the leaf size in control and transgenic lines. All data represent averages of three biological replicates, error bars indicate SD. Statistical significance was determined using the Student’s *t*-test (*p < 0.05, **p < 0.01).

After obtaining the OE and interference lines of *S. miltiorrhiza*, the plantlet seedlings were cultured for 60 days, transplanted into soil media as part of a hydroponic cultivation system, and then cultivated for another 60 days. There were obvious morphological differences in the OE and interference SmWD40-170 transgenic lines. The results showed that the growth state of OE-3 line was better than the CK line and had higher biomass accumulation in both leaves and roots. In contrast, the RNAi line was shorter, and the growth state was poorer (Figures 8A–F). Statistical data was consistent with the phenotype. Compared with the CK, the OE lines had the advantages of root length. The feature was more obvious compared to those in the RNAi lines (Figures 8G–I,M). In addition, morphological changes were observed in the leaves. There was no significant difference in leaf size between OE with CK lines, however, the RNAi lines had smaller leaves with curled leaf edges (Figures 8J–L,N). These results indicate that the SmWD40-170 protein is necessary for the growth and development of *S. miltiorrhiza*.

## Discussion

The plant-specific TIFY family plays vital roles in the growth, development, and secondary metabolism of plants (Vanholme et al., 2007; Ye et al., 2009; Bai et al., 2011; Zhang et al., 2012, 2015; Zhu et al., 2013; Saha et al., 2016). Significant progress has been made toward identifying and characterizing the *TIFY* genes in various species. In the present study, we identified and analyzed the TIFY families in *S. miltiorrhiza*. This species contains 10 *SmJAZ*s, 3 *SmZML*s, 1 *SmTIFY*, and 1 *SmPPD* genes (Table 1). Similar to other species, the SmJAZ proteins have two typical domains, N-terminal TIFY and C-terminal Jas. The TIFY domain participates in homomeric and heteromeric interactions, or in the interactions between JAZ proteins and MYC TFs (Bai et al., 2011). The Jas motif interacts with COI1, bHLH, or R2R3-MYB members (Chico et al., 2008; Browse, 2009). The SmZML proteins not only have these two domains, but also contain a CCT domain, similar to the Jas domain, and a GATA zinc finger. In contrast, SmPPD also has a PPD domain at its N-terminal (Figure 2A). SmJAZ1, SmJAZ2, SmJAZ5, and SmJAZ6 each carry an EAR-like motif (Figure 2A), which also exists in plant AUX/IAA proteins and functions as a binding motif for the regulator repressor TOPLESS. This suggests that these four proteins have different functions that are more critical than those of the other SmTIFY proteins (Szemenyei et al., 2008). Although all SmJAZ proteins could be clustered into the four groups previously described for other species (Figure 1), the high degree of variability among the sequences within this subfamily, suggests that these proteins have had a possible divergence in functions.

Members of the SmTIFY family are diverse in their exons and introns (Figure 2B), implying an important evolutionary role for their gene structure (Bai et al., 2011). Sequence alignments and phylogenetic analysis of the SmTIFY proteins indicated that almost all could be classified into the same four major groups with TIFY proteins from other species with the exception is for the rice members, from which OsPPD is missing. Screening the TIFY proteins from the different species has demonstrated that they only occur among Embryophyta (land plants) and that both group I (TIFY proteins with a C2C2-GATA domain) and group II (TIFY proteins without C2C2-GATA domain) of TIFY proteins are present in the liverworts (Marchantiophyta), which are considered primitive land plants (Vanholme et al., 2007). This suggests that the TIFY family was essential for the emergence of land plants during a series of evolutionary adaptations that increased the complexity of plant structure. This probably also enhanced the ability of those plants to respond to adverse environmental conditions. In contrast to many plant-specific gene families, obvious diversification has occurred between monocot and dicot species (Bai et al., 2011). Therefore, the plant *TIFY* genes may be derived from common ancestors that existed before the divergence of monocot and dicot species.

SmTTG1, a member of the WD40 protein family, has been shown to promote the accumulation of salvianolic acids in *S. miltiorrhiza* (Li et al., 2018). In the present study, we screened a WD40 protein, which participates in both salvianolic acids and tanshinones biosynthesis, except in drought stress responses (Liu et al., 2020). In the OE lines, the contents of the salvianolic acids and tanshinones were significantly increased, by approximately two fold, and the RNAi lines were expectedly decreased by at least 50%, which is consistent with the changes in the biosynthesis of the enzyme genes in the transgenic lines (Figures 6, 7). Notably, all 14 key genes selected were induced by SmWD40-170. However, the WD40 protein is unable to directly regulate enzyme genes, and probably functions together with MYB and bHLH proteins to generate the MBW complex. In future research, we will focus on investigating the interaction partners of the SmWD40-170 protein. In addition, some obvious phenotypic changes were observed in the transgenic lines. Compared with the CK line, the RNAi lines presented underdeveloped roots, smaller leaves, and curled leaf edges, but the OE lines showed the opposite traits, with well-developed roots (Figure 8). In particular, the roots of OE lines were redder than those of the CK and RNAi lines. These results are consistent with the content determination of tanshinones in the transgenic and CK lines (Figure 6), and in accordance with the fact that more tanshinones were gathered in the redder roots of *S. miltiorrhiza* (Wang et al., 2014). It is great significance when aiming to improve the quality of *S. miltiorrhiza*. SmWD40-170 not only regulates secondary metabolism, but also affects growth and development, however, links between growth and secondary metabolite accumulation warrant further investigation.

**FIGURE 9:**
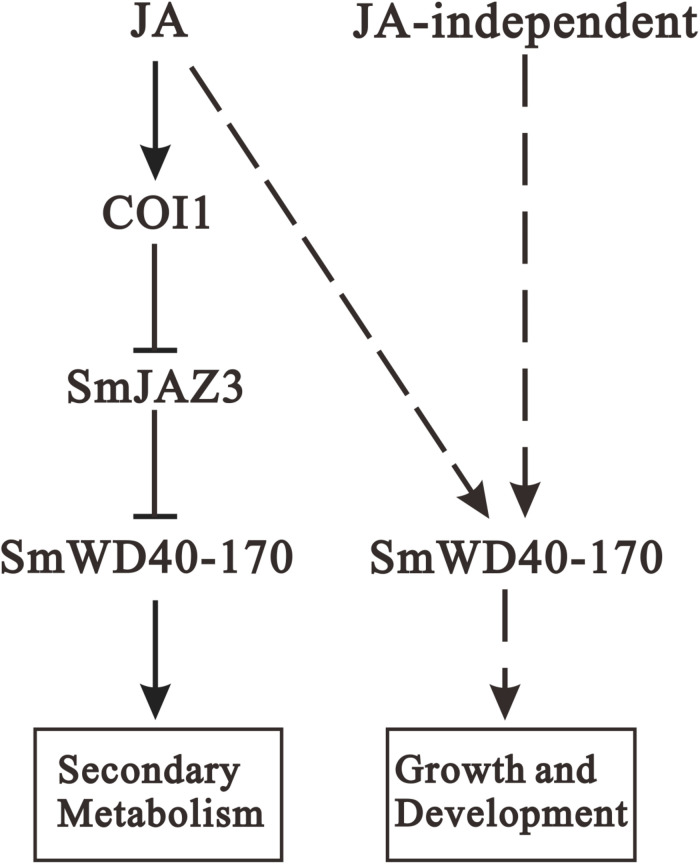
Proposed module of the roles of JA in regulating secondary metabolites biosynthesis as well as growth and development. When treating with exogenous JA, the complex formation between the JA-Ile and COI1 promotes SmJAZ3 degradation via the 26S proteasome, and release the positive regulators such as WD40, then enhances the activities of enzymes to promote secondary metabolites biosynthesis. In addition, SmWD40-170 protein may regulate growth and development through JA-dependent or JA-independent pathway. Arrows, positive regulation; blunt ends, negative regulation; dotted line, uncertified process.

Jasmonic acid has been shown to enhance the accumulation of secondary metabolites and promote growth and development in *S. miltiorrhiza* (Xiao et al., 2010; Gu et al., 2012; Ge et al., 2015; Shi et al., 2020), where SmCOI1 also plays a critical role (Chen et al., 2018). JAZ proteins are the targets of the SCF^*COI*1^ complex and function as key components of the JA signaling pathway (Shi et al., 2016; Pei et al., 2018), however, the mechanisms underlying JAZ-regulated repression events remain unclear for *S. miltiorrhiza*. Since JAZ proteins contain no DNA-binding domain, JAZs might affect gene expression and metabolite accumulation through their interactions with target genes (Cheng et al., 2011). It has been reported that a Jas motif of the JAZ proteins, participates in the protein–protein interactions with the MYB, bHLH, and other TFs (Withers et al., 2012). In the present study, we found that a WD40 protein interacts with the Jas motif of SmJAZ3 using Y2H screening, and determine the interaction relationship between the SmJAZ3 and SmWD40-170. SmJAZ3 has been reported to act as a repressive transcriptional regulator in tanshinones biosynthesis regulation (Shi et al., 2016), while SmWD40-170 responds to drought stress by regulating ABA- and H_2_O_2_- induced stomal movement in *S. miltiorrhiza* (Liu et al., 2020). In the present study, we speculated that SmWD40-170 regulates the accumulation of secondary metabolites by interacting with SmJAZ3 in *S. miltiorrhiza*, which brings insights into the molecular mechanism of SmJAZ3 regulates tanshinone biosynthesis. We propose that SmJAZ3 protein interacts with SmWD40-170 in the JA signaling pathway and plays a vital role in the accumulation of secondary metabolites (Figure 9), which provides essential information for further exploring the mechanisms by which JA regulates the biosynthesis and accumulation of secondary metabolites in *S. miltiorrhiza*.

## Conclusion

This study identified and analyzed 15 SmTIFY family members. Phylogenetic analysis suggested that the SmTIFY proteins could be clustered into four groups. RT-qPCR results showed that most of the *SmTIFY* genes responded to the MeJA treatments. Our analysis illustrated diversity in the *cis*-elements among the SmTIFY members, indicating that these genes have important roles in several hormone signaling pathways and stress responses, and may thus be applied to increase the production of valuable plant compounds. Furthermore, a novel interaction partner of SmJAZ3 was screened and physical interactions between SmJAZ3 and SmWD40-170 was also demonstrated in this study, which suggests a potential regulation mechanism for the SmJAZ involved in the JA signaling pathway. Subsequently, genetic assays showed that SmWD40-170 positively induced the accumulation of salvianolic acids and tanshinones, and promoted plant growth and development. Collectively, our research lays a foundation for future investigations into the mechanism of the hormone signal regulation network among the SmTIFY family members.

## Data Availability Statement

The original contributions presented in the study are included in the article/Supplementary Material, further inquiries can be directed to the corresponding authors.

## Author Contributions

LL, XC, and ZW designed the experiments. LL, YH, and YL performed the experiments. YL, WM, and DW contributed analytical tools and provided technical support. LL and YH wrote the manuscript. BL, XC, and ZW promoted the manuscript. All authors revised and approved the manuscript.

## Conflict of Interest

The authors declare that the research was conducted in the absence of any commercial or financial relationships that could be construed as a potential conflict of interest.

## Footnotes

1 http://www.arabidopsis.org

2 http://rice.plantbiology.msu.edu

3 http://www.genoscope.cns.fr

4 https://www.ncbi.nlm.nih.gov/Structure/cdd/wrpsb.cgi

5 http://pfam.xfam.org/

6 http://www.ebi.ac.uk/Tools/hmmer/

7 http://www.ebi.ac.uk/interpro/search/sequence-search

8 http://smart.embl.de/

9 http://www.megasoftware.net/

10 http://web.expasy.org/protparam/

11 http://www.genscript.com/wolf-psort.html

12 http://gsds.gao-lab.org/

13 http://meme-suite.org/

14 http://bioinformatics.psb.ugent.be/webtools/plantcare/html/

## Supplementary Material

Supplementary Figure 1 Screening of cDNA library using SmJAZ3-jas as bait. SmJAZ3 **(A)** and SmJAZ3-jas **(B)** bait vectors construction for screening yeast two-hybrid library. **(C)** Transcriptional activity analysis of BD-SmJAZ3 in yeast. **(D)** Bait bacteria with library bacteria forming yeast conjugates. **(E)** Part of the positive clones of yeast two-hybrid screening. **(F)** Agarose gel electrophoresis examine the PCR products for part of the yeast plaque.

Supplementary Figure 2 MRM maps of rosmarinic acid, salvianolic acid B, tanshinone IIA, and cryptotanshinone standard and sample.

Supplementary Table 1 Primer sequences used for RT-qPCR.

Supplementary Table 2 Primers for PCR and vectors construction.

Supplementary Table 3 Part of *cis*-elements in *SmTIFY* family genes. A series of *cis*-elements involved in light response, the biotic and abiotic stress response, phytohormone (abscisic acid, auxin, salicylic acid, gibberellin, ethylene, MeJA) response, circadian control, MYB binding site, G-box, endosperm and meristem expression, and fungal elicitor response were identified.

Supplementary Table 4 Yeast library screening with SmJAZ3-Jas-BD as bait protein, NCBI BlastX results.

## Abbreviations

JA: jasmonate
MeJA: methyl jasmonate
JAZ: jasmonate ZIM
WD40 protein: WD40 repeat-containing protein
TF: transcription factor
CDS: coding sequence
RT-qPCR: quantitative reverse transcription PCR
LC/MS: liquid chromatography/mass spectrometry
CK: control check
OE: overexpression
RNAi: ribonucleic acid interference
YFP: yellow fluorescent protein
GFP: green fluorescent protein
Y2H: yeast two-hybrid
BiFC: bimolecular fluorescence complementation
EAR: ethylene-responsive element binding factor-associated amphiphilic repression
SA: salicylic acid
MBS: MYB binding site
HSE: heat shock element
DRE: dehydration responsive element
WUN: wound.
