# Supplementary figures and images for: Genome-Wide Identification of the TIFY Family in Salvia miltiorrhiza Reveals That SmJAZ3 Interacts With SmWD40-170, a Relevant Protein That Modulates Secondary Metabolism and Development
Source: Front Plant Sci. 2021 Feb 18;12:630424. doi: 10.3389/fpls.2021.630424 (PMC7930841; doi:10.3389/fpls.2021.630424)

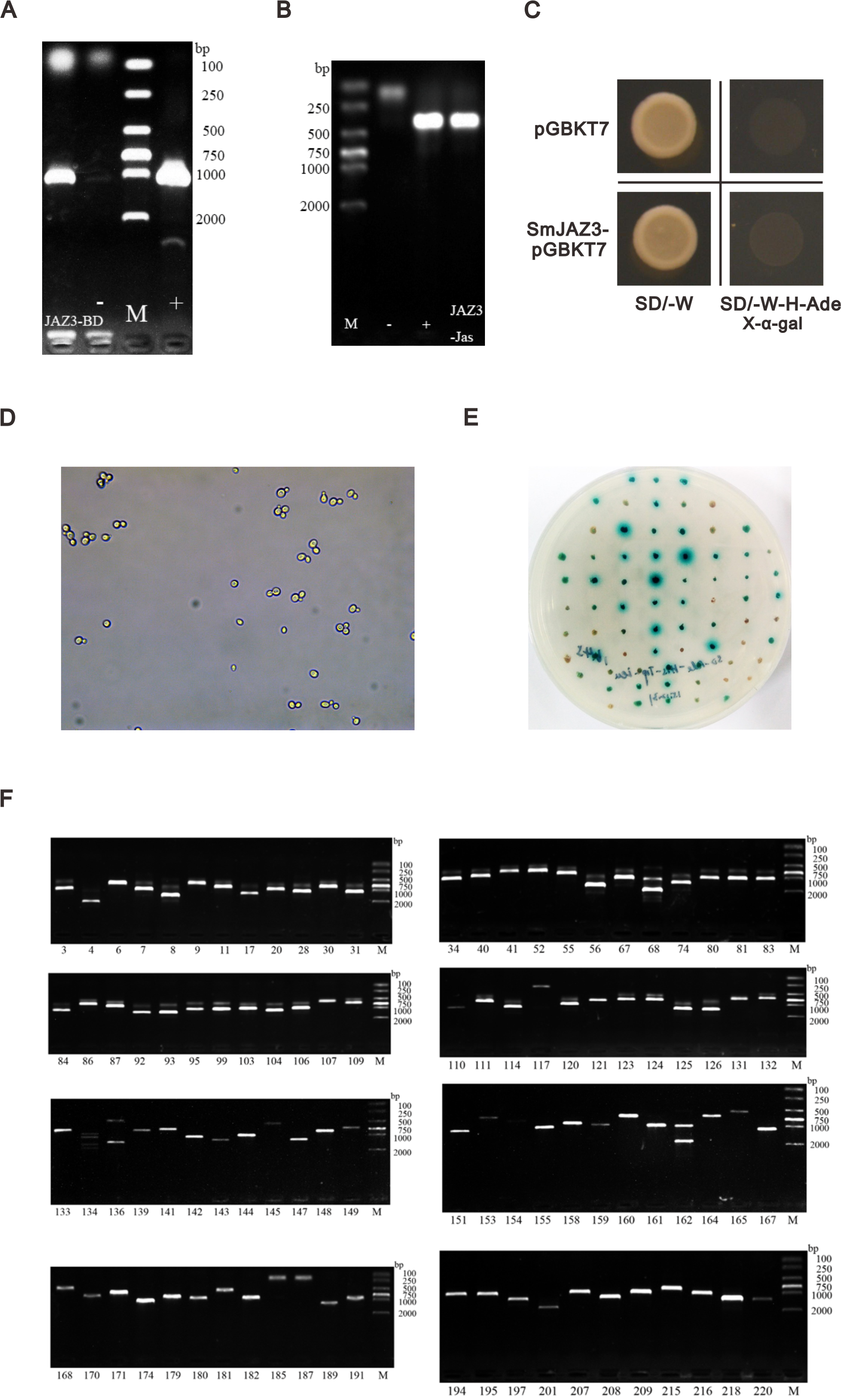

Supplement: Supplementary Figure 1 — Screening of cDNA library using SmJAZ3-jas as bait. SmJAZ3 (A) and SmJAZ3-jas (B) bait vectors construction for screening yeast two-hybrid library. (C) Transcriptional activity analysis of BD-SmJAZ3 in yeast. (D) Bait bacteria with library bacteria forming yeast conjugates. (E) Part of the positive clones of yeast two-hybrid screening. (F) Agarose gel electrophoresis examine the PCR products for part of the yeast plaque. [file Image_1.TIF]

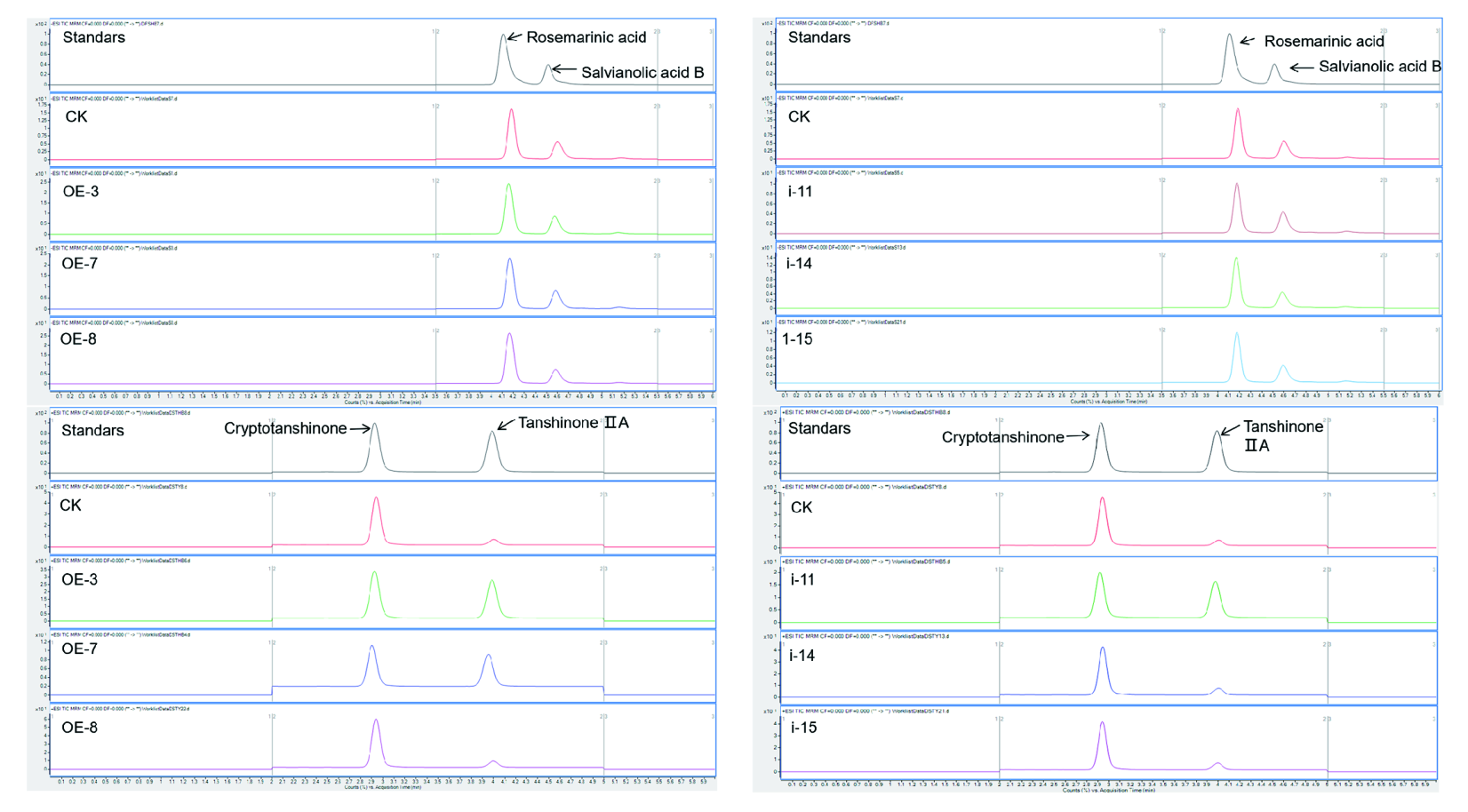

Supplement: Supplementary Figure 2 — MRM maps of rosmarinic acid, salvianolic acid B, tanshinone IIA, and cryptotanshinone standard and sample. [file Image_2.TIF]
