# Supplementary material for: Genome-Wide Identification of the TIFY Family in Salvia miltiorrhiza Reveals That SmJAZ3 Interacts With SmWD40-170, a Relevant Protein That Modulates Secondary Metabolism and Development
Source: Front Plant Sci. 2021 Feb 18;12:630424. doi: 10.3389/fpls.2021.630424 (PMC7930841; doi:10.3389/fpls.2021.630424)
Supplement: Supplementary Table 1 — Primer sequences used for RT-qPCR. [file Table_1.docx]

**Table S1** Primer sequences used for RT-qPCR

| Primer Sequence | Primer Sequence |
| --- | --- |
| SmJAZ1-RTF AGGTCCAACTTCTCGCAAAC  SmJAZ1-RTR ATCGTCATCTGCCCAATTTC  SmJAZ2-RTF AATCAAAGGGGTTCCTACGG  SmJAZ2-RTR CCGGAAAGTCGTTGAAAACT  SmJAZ3-RTF CCGTTGGAACCACTGATTTT  SmJAZ3-RTR AATGTGCATTTCCAGCCAAT  SmJAZ4-RTF AATCCTTTCTGCCCCTTAGC  SmJAZ4-RTR TATCCGCAGATACGTTGTCG  SmJAZ5-RTF GCCAATTGCAAGGAGATCAT  SmJAZ5-RTR CGACTTGAGGTTCTCGAGGT  SmJAZ6-RTF TCCAACGCCTCTAAGGAGAA  SmJAZ6-RTR CCTTTGAAGAAGAGCCTGGA  SmJAZ7-RTF CCACCCAATTACAACGCTTC  SmJAZ7-RTR TGCCTGCAACCATTCCATCCAG  SmJAZ8-RTF GTAACGCCTTCGGTCTCCTT  SmJAZ8-RTR TTCCTGCTCGATTTTTCCTC  SmJAZ9-RTF TCGAAAGTTCACATGCAAGC  SmJAZ9-RTR TGACATGCCAGCGATCTTAG  SmJAZ10-RTF AGGTGGTGAAGAGCGTGATT  SmJAZ10-RTR GCGTGCAGAGACGTCAAATA  SmTIFY8-RTF ATATTTTATGGGGGCCAAGC  SmTIFY8-RTR AGCTCCCTCAGCATAGCAAG  SmPPD-RTF GACTGCCGCCGTTACCTCAA  SmPPD-RTR CCGGCGTTGTTTCGAGGAGT  SmZML1-RTF CGTGAAATGCCTCCTAACCT  SmZML1-RTR TTCCGTTGCATCCTTAGAGC  SmZML2-RTF GACCAGCTCACCCTTTCCTT  SmZML2-RTR TTTGAGGCTGACTCGACCTT  SmZML3-RTF AGCCAGCCTACTGTTTCTCG  SmZML3-RTR TTCCGGGATTCCAATTATCA | *Smβ-Actin*-RTF AGGAACCACCGATCCAGACA  *Smβ-Actin*-RTR GGTGCCCTGAGGTCCTGTT  SmPAL-RTF GATAGCGGAGTGCAGGTCGTAC  SmPAL-RTR CGAACTAGCAGATTGGCAGAGG  Sm4CL-RTF TCACCCATGCCGGATTCGAG  Sm4CL-RTR AGATCGCGCCGATGAAGGAG  SmC4H-RTF CCAGGAGTCCAAATAACAGAGCCG  SmC4H-RTR GCCACCAAGCGTTCACCAAGAT  SmTAT-RTF CGAGCAGGGATGGGAGGTTG  SmTAT-RTR GCCTCTTGGCTGTCTCAGCA  SmHPPR-RTF TGACTCCAGAAACAACCCACATT  SmHPPR-RTR CCCAGACGACCCTCCACAAG  SmRAS-RTF CCAAAGTCAATTATGCCAAGGG  SmRAS-RTR GTCGGATAGGTGGTGCTCGT  SmCYP98A14-RTF CCAATCCTACGGCCCGATCC  SmCYP98A14-RTR GCCGTCTCTGCTGAGCTTGA  SmDXS-RTF GGGTCACCTGGGATCAAGCC  SmDXS-RTR GGTGGCCAACATCCCACAGA  SmHMGR-RTF CATGCCTCAACCTGCTGGGA  SmHMGR-RTR ATCGCCGACATGAGCGAGAG  SmFPPS-RTF TCCAGGGCCTTTACAACCAGC  SmFPPS-RTR TTCATCGCCGCATTGTTCAGT  SmGGPPS-RTF CATTTCCTCCCACGGCGTCT  SmGGPPS-RTR CGTCGTCAAGCGCCTTGTTC  SmCPS-RTF AGAGAATCGCGTGGGCCAAA  SmCPS-RTR CGCCGTTTGTGTCGTTGAGG  SmKSL-RTF GGAGCGATGACACGGCACTA  SmKSL-RTR GCCGGTCAACCATGGAGACAT  CYP76AH1-RTF AACGCTTCCTCGATAACAAGAT  CYP76AH1-RTR TTATGAACCAGAGTCGCAGTAG |
