# Supplementary material for: Genome-Wide Identification of the TIFY Family in Salvia miltiorrhiza Reveals That SmJAZ3 Interacts With SmWD40-170, a Relevant Protein That Modulates Secondary Metabolism and Development
Source: Front Plant Sci. 2021 Feb 18;12:630424. doi: 10.3389/fpls.2021.630424 (PMC7930841; doi:10.3389/fpls.2021.630424)
Supplement: Supplementary Table 2 — Primers for PCR and vectors construction. [file Table_2.docx]

**Table S2** Primers for PCR and vectors construction

| **Primers** | **Sequences (5’-3’)** |
| --- | --- |
| *SmJAZ3*-F | ATGGGTTTGTCTGTGAAGCAGGAGG |
| *SmJAZ3*-R | TCAATTGGCAGCTGGAACTGGACA |
| *SmJAZ3*-Jas-F(for Y2H screening) | GTCTCCCAGTCTGCTAATAAGCCATC |
| *SmJAZ3*-Jas-R(for Y2H screening) | TCAATTGGCAGCTGGAACTGGACA |
| BD-*SmJAZ3*-GF | GGGGACAAGTTTGTACAAAAAAGCAGGCTTC ATGGGTTTGTCTGTGAAGCAGGAGG |
| BD-*SmJAZ3*-GR | GGGGACCACTTTGTACAAGAAAGCTGGGTC  TCAATTGGCAGCTGGAACTGGACA |
| BD-*SmJAZ3*-Jas-GF | GGGGACAAGTTTGTACAAAAAAGCAGGCTTC GTCTCCCAGTCTGCTAATAAGCCATC |
| BD-*SmJAZ3*-Jas-GR | GGGGACCACTTTGTACAAGAAAGCTGGGTC  TCAATTGGCAGCTGGAACTGGACA |
| BD-*SmJAZ3*NT-GF | GGGGACAAGTTTGTACAAAAAAGCAGGCTTC  ATGGAGAGAGATTTCATGGGTT |
| BD-*SmJAZ3*NT-GR | GGGGACCACTTTGTACAAGAAAGCTGGGTC  TGATAGAAAGCTTGCAGATCCT |
| BD-*SmJAZ3*Jas-GF (for Y2H assay) | GGGGACAAGTTTGTACAAAAAAGCAGGCTTC  TCAGACACTGTGCCTCAGTTTC |
| BD-*SmJAZ3*Jas-GR (for Y2H assay) | GGGGACCACTTTGTACAAGAAAGCTGGGTC  ATAAGGTGAAGCACTGATCACC |
| BD-*SmJAZ3*CT-GF | GGGGACAAGTTTGTACAAAAAAGCAGGCTTC  GGTGAGTGTCAATCTGGGGA |
| BD-*SmJAZ3*CT-GR | GGGGACCACTTTGTACAAGAAAGCTGGGTC  TCAATTGGCAGCTGGAACT |
| AD-*SmWD40-170*-GF | GGGGACAAGTTTGTACAAAAAAGCAGGCTTC ATGAAACTAGCGCCATTGAACAC |
| AD-*SmWD40-170*-GR | GGGGACCACTTTGTACAAGAAAGCTGGGTC  TCAGGAATATTTATAGAACGATACACTC |
| YC-*SmJAZ3*-GF | GGGGACAAGTTTGTACAAAAAAGCAGGCTTC ATGGGTTTGTCTGTGAAGCAGGAGG |
| YC-*SmJAZ3*-GR | GGGGACCACTTTGTACAAGAAAGCTGGGTC  ATTGGCAGCTGGAACTGGACA |
| YN-*SmWD40-170*-GF | GGGGACAAGTTTGTACAAAAAAGCAGGCTTC ATGAAACTAGCGCCATTGAACAC |
| YN-*SmWD40-170*-GR | GGGGACCACTTTGTACAAGAAAGCTGGGTC  GGAATATTTATAGAACGATACACTC |
