# Supplementary material for: Genome-Wide Identification of the TIFY Family in Salvia miltiorrhiza Reveals That SmJAZ3 Interacts With SmWD40-170, a Relevant Protein That Modulates Secondary Metabolism and Development
Source: Front Plant Sci. 2021 Feb 18;12:630424. doi: 10.3389/fpls.2021.630424 (PMC7930841; doi:10.3389/fpls.2021.630424)
Supplement: Supplementary Table 3 — Part of cis-elements in SmTIFY family genes. A series of cis-elements involved in light response, the biotic and abiotic stress response, phytohormone (abscisic acid, auxin, salicylic acid, gibberellin, ethylene, MeJA) response, circadian control, MYB binding site, G-box, endosperm and meristem expression, and fungal elicitor response were identified. [file Table_3.docx]

**Table S3** Part of *cis*-elements in *SmTIFY* family genes. A series of *cis*-elements involved in light response, the biotic and abiotic stress response, phytohormone (abscisic acid, auxin, salicylic acid, gibberellin, ethylene, MeJA) response, circadian control, MYB binding site, G-box, endosperm and meristem expression, and fungal elicitor response were identified

| ***Cis*-element** | **Function** | **Gene** | |
| --- | --- | --- | --- |
| ABRE | cis-acting element involved in the abscisic acid responsiveness | SmJAZ1/2/3/5/6/8, SmPPD, SmZML1/3 | |
| P-box | gibberellin-responsive element | SmJAZ1, SmTIFY8/PPD, SmZML3 | |
| CGTCA-motif | cis-acting regulatory element involved in the MeJA-responsiveness | SmJAZ1/2/5/6/7, SmZML2 | |
| TGACG-motif | cis-acting regulatory element involved in the MeJA-responsiveness | SmJAZ2/5/6/7, SmZML2 | |
| TGA-element | auxin-responsive element | SmJAZ1/3/2/5/7, SmTIFY8, | |
| TCA-element | cis-acting element involved in salicylic acid responsiveness | SmJAZ2/3/4/5/6/7/8/9/10, SmPPD, SmZML1/3 | |
| ERE | ethylene-responsive element | SmJAZ6/10, SmZML1/3 | |
| GARE-motif | gibberellin-responsive element | SmJAZ7/8, SmTIFY8/PPD, SmZML3 | |
| TC-rich repeats | cis-acting element involved in defense and stress responsiveness | SmJAZ1/2/3/4/5/6/7/8/9/10, SmTIFY8, SmZML2/3 | |
| HSE | cis-acting element involved in heat stress responsiveness | SmJAZ4/5/6/7/8/9/10, SmTIFY8/PPD, SmZML1/3 | |
| LTR | cis-acting element involved in low-temperature responsiveness | SmJAZ5/10, SmZML1 | |
| MBS | MYB binding site involved in drought-inducibility | SmJAZ2/4/5/6/7/9/10, SmTIFY8/PPD, SmZML1/3 | |
| MRE | MYB binding site involved in light responsiveness | | SmJAZ9/10, SmTIFY8, |
| MBSI | MYB binding site involved in flavonoid biosynthetic genes regulation | SmPPD | |
| WUN-motif | wound-responsive element | SmJAZ10 | |
| C-repeat/DRE | regulatory element involved in cold- and dehydration-responsiveness | SmJAZ10 | |
| Skn-1_motif | cis-acting regulatory element required for endosperm expression | SmJAZ1/3/4/5/6/7/8/10, SmTIFY8/PPD, SmZML1 | |
| GCN4_motif | cis-regulatory element involved in endosperm expression | SmJAZ6/8/10, SmTIFY8, SmZML2 | |
| ARE | cis-acting regulatory element essential for the anaerobic induction | SmJAZ1/2/3/6/7/10, SmTIFY8/PPD, SmZML2/3 | |
| CAT-box | cis-acting regulatory element related to meristem expression | SmJAZ4/2, | |
| CCGTCC-box | cis-acting regulatory element related to meristem specific activation | SmJAZ7/8/9/10, SmTIFY8, SmZML2/3 | |
| OCT | cis-acting regulatory element related to meristem specific activation | SmJAZ7, SmZML3 | |
| circadian | cis-acting regulatory element involved in circadian control | SmJAZ3/4/8, SmTIFY8, SmZML1/3 | |
| Box-W1 | fungal elicitor responsive element | SmJAZ1/4/7/10, SmPPD | |
| CCAAT-box | MYBHv1 binding site | SmJAZ2, SmZML3 | |
| O2-site | cis-acting regulatory element involved in zein metabolism regulation | SmJAZ2/5, | |
| MSA-like | cis-acting element involved in cell cycle regulation | SmZML3 | |
| G-box | cis-acting regulatory element involved in light responsiveness | SmJAZ1/2/4/5/6/7/8/9/10, SmTIFY8/PPD, SmZML1/2/3 | |
| I-box | part of a light responsive element | SmJAZ1/4/2/7, SmTIFY8/PPD, SmZML3 | |
| HD-Zip 1 | element involved in differentiation of the palisade mesophyll cells | SmTIFY8 | |
| HD-Zip 2 | element involved in the control of leaf morphology development | SmTIFY8 | |
