# Supplementary material for: Genome-Wide Identification of the TIFY Family in Salvia miltiorrhiza Reveals That SmJAZ3 Interacts With SmWD40-170, a Relevant Protein That Modulates Secondary Metabolism and Development
Source: Front Plant Sci. 2021 Feb 18;12:630424. doi: 10.3389/fpls.2021.630424 (PMC7930841; doi:10.3389/fpls.2021.630424)
Supplement: Supplementary Table 4 — Yeast library screening with SmJAZ3-Jas-BD as bait protein, NCBI BlastX results. [file Table_4.docx]

**Table S4** Yeast library screening with SmJAZ3-Jas-BD as bait protein, NCBI BlastX results

| number | name | number | name |
| --- | --- | --- | --- |
| 3/17/80/92/93/143/170/179/195/207/220 | SMLII [Salvia miltiorrhiza] | 114 | APD protein 2 |
| 6 | MYC2 | 121 | polyphenol oxidase |
| 7/28/31/34/67 | phospholipase C | 123/141 | epidermal patterning factor protein 4 |
| 8/136 | WD40-170 | 125 | （DUF2146）LOC105170220/SMG8 |
| 11 | adenosine deaminase | 126 | Porin3 superfamily |
| 20/110 | cysteine protease 15A | 132 | DUF2741 |
| 30 | aspartic protease X2 | 133 | Diacylglycerol Acyltransferase |
| 40/55/103/106/111/120/142/144/158/160/161/171/194 | vanillin synthase | 134 | LOC104900590 |
| 41/52/117/124 | aspartic protease | 139 | lectin F511_12330 |
| 56 | diphtheria synthase POPR | 151 | L- ascorbate peroxidase |
| 68 | LOC105951806 | 153 | SOVF-077440 |
| 74/208 | cysteine protease 3 | 155 | cysteine protease（DCAR_028336） |
| 81/83/95/99/209/216 | lectin F511_19809 | 162/197 | galactinol synthase |
| 84 | LOC105168160 | 164/167/180 | GAPDH |
| 86 | phosphoglucomutase | 174 | LOC105955094 |
| 87/215 | RICIN Super | 182 | Cytochrome b6f |
| 104 | LOC105168160 | 189 | acid phosphatase |
| 107 | Gal4 activates peptides | 191 | [ubiquitin fusion proteins](https://kns.cnki.net/KNS8/Detail/RedirectScholar?flag=TitleLink&tablename=SSJD_01&filename=SSJDFC69E28E125AE745683F212E60D294E2) |
|  |  |  |  |
